# Bioethanol Production From Hydrolyzed Lignocellulosic After Detoxification Via Adsorption With Activated Carbon and Dried Air Stripping

**DOI:** 10.3389/fbioe.2018.00107

**Published:** 2018-07-31

**Authors:** Wagner Artifon, Charline Bonatto, Eduarda R. Bordin, Suzana F. Bazoti, Adriana Dervanoski, Sérgio L. Alves, Helen Treichel

**Affiliations:** ^1^Laboratory of Microbiology and Bioprocess, Department of Environmental Science and Technology, Federal University of Fronteira Sul, Erechim, Brazil; ^2^Environmental Engineering, Federal University of Fronteira Sul, Erechim, Brazil; ^3^Laboratory of Biochemistry and Genetics, Federal University of Fronteira Sul, Chapecó, Brazil

**Keywords:** bioethanol, acid acetic, detoxification, dried air stripping, adsorption

## Abstract

Bioethanol production has been presented as an alternative for supplying energy demand and minimizing greenhouse gases effects. However, due to abrasively conditions employed on the biomass during pretreatment and hydrolysis processes, inhibitors for fermentation phase such as acetic acid and others can be generated. Based on this problem, the aim of this work was to evaluate the adsorption of acetic acid on microporous activated carbon and investigate the stripping of the same component with dried air. For adsorption process, three concentrations of acetic acid (5, 10, and 20%) were analyzed by adsorption kinetics and adsorption isotherms (Langmuir and Freundlich models). Pseudo-second order model showed to fit better when compared to Pseudo-first order model. The Intraparticle Diffusion model presented the first phase of the adsorption as the regulating step of the adsorption process. The Langmuir model showed the best fitting, and the maximum capacity of adsorption was found as 128.66 mg.g^−1^. For stripping procedure an apparatus was set in order to insert dried air by a diffusor within the solution in study. Increasing temperature showed to be determinant on augmenting acetic acid evaporation in 2.14 and 6.22 times for 40 and 60°C when comparing it to 20°C. The application of the pickling process for removal of fermentation inhibitors in sugarcane bagasse hydrolyzed allowed the production 8.3 g.L^−1^ of ethanol.

## Introduction

Biofuels have been stated as the renewable source of energy substituting fossil fuels. Once produced by biomass, it does not promote an augmenting in greenhouse gases concentration because it can again be assimilated by ecosystem during photosynthesis process (Rivera-Méndez et al., 2017). Brazil has been presented as a leader in this scenario, mainly due to the robust and efficient ethanol production from sugarcane (Moreira et al., 2016). Besides that, Brazil also presents a potential to be a great producer of bioethanol due to the large generation of sugarcane bagasse along with others lignocellulosic materials (Carvalho et al., 2016).

However, lignocellulosic ethanol is produced from cheap feedstocks; biomass requires several physical and chemical procedures to be unstructured in its three main polymers: cellulose, hemicellulose and lignin before releasing sugar (Kundu et al., 2015; De Bhowmick et al., 2018). Pretreatment and hydrolysis are necessary steps that are generally conducted under abrasive process where toxic components can be engendered and inhibit the yeast during fermentation process (Diaz et al., 2018; Muharja et al., 2018). Acetic acid, generated by hemicellulose degradation, is an inhibitor that presents relevance in the medium due to its capacity of pass through the membrane of the fungal cell and affect its metabolism. Others inhibitors produced during process are also responsible for deleterious effects on the yeast (Huang et al., 2011; Diaz et al., 2018). Therefore, the detoxification of the hydrolyzed of biomass is important for improving bioethanol yield during fermentation process (Zhang et al., 2015).

Activated carbon have been defined as the best adsorbent for organic compounds due to its porous structure, aggregating to the material a high surface area and a great adsorbability (Gamal et al., 2018). Even applied in a variety of studies and being a cost effective adsorbent (Li et al., 2016), adsorption pairs (adsorbent-adsorbate) with activated carbon still demand investigation to improve many process (Sellaoui et al., 2015; Singh and Kumar, 2016). Therefore, setting adsorption kinetics and isotherms is essential for promoting the understanding in the relation between pairs.

In this way, the objective of this study was to evaluate adsorption of acetic acid by a microporous activated carbon as well as set isotherms and kinetic characteristics between this pair. At the same time, it is intended to investigate stripping of the same component from a similar solution by dried air. The condition that resulted in a hydrolyzate with a lower concentration of acetic acid was tested in an alcoholic fermentation.

## Materials and methods

### Adsorption

A microporous activated carbon in powder form (Vetec) and glacial acetic acid were used in this study. Kinetics and equilibrium curves between solid and liquid phase were performed in order to characterize the adsorption of acetic acid by activated carbon. Three kinetics with different concentrations of acetic acid, 5, 10, and 20% (w/w), were conducted in Erlenmeyer 250 mL. 10 g of activated carbon was added to 200 mL of acetic acid solution in the concentrations in study. The kinetic time was set as 1 h, occurring in shaker, with an agitation of 120 rpm at ambient temperature. Aiming to check the saturation time of the activated carbon, eight samples were taken during the kinetics. All experiments were based on previous tests. For equilibrium curves four temperatures, 20, 40, 60, and 70°C, and eight concentrations, 2, 4, 6, 8, 10, 12, 14, and 16% (w/w) were tested. Forty milliliter of acetic acid solution at each concentration were added to Erlenmeyer 125 mL along with 2 g of activated carbon. Samples temperatures were stabilized in thermostatic bath before half hour in shaker at each temperature in study with 120 rpm as operating agitation. In this experiment, all samples taken for measurement were filtrated with filter paper and 5 mL of permeate was measured by neutralization method with sodium hydroxide (NaOH). Experiments were carried out twice.

### Stripping

**Figure 1:**
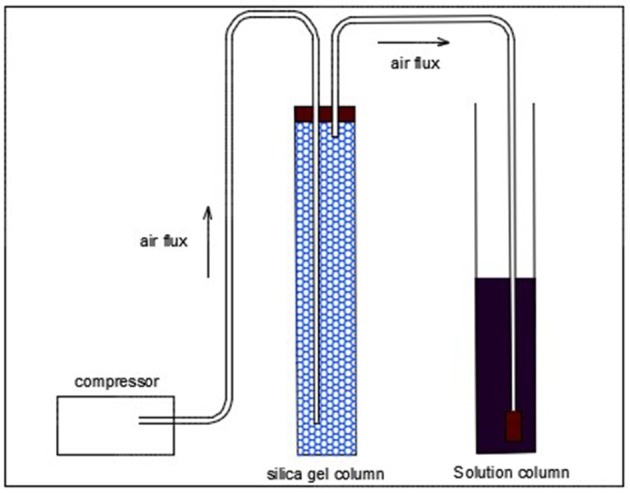
Representation of the stripping process.

An apparatus was developed to run the experiments as Figure 1. It consists of a compact compressor which pumps air through a silica column, the dried air resultant is inserted into the solution in test by a micro bubble diffusor. The air leaves the silica column with a relative humidity of 20% and improves desorption of components from the liquid to the gas phase. Three kinetics at different temperatures were conducted in order to investigate the capacity of dried air on carrying acetic acid out from the solution. 200 g of an acetic acid solution of 10% (w/w) were added to the column along with the diffusor which releases a flux of 0.82 L.min^−1^ of air. Experiments were carried out within a thermostatic bath to maintain the system at the required temperatures of 20, 40, and 60°C. The kinetics were performed during 30 h, collecting samples of 5 mL and checking weigh loss at each 6 h. Experiments were based on previous tests. Samples concentration were measured by neutralization with sodium hydroxide (NaOH).

### Detoxification hydrolysate lignocellulosics by dried air stripping

#### Hydrolysate

The cellulosic hydrolysate used as substrate was kindly provided by the CTC-Centro de Tecnologia Canavieira, located in São Paulo, Brazil. The broth was obtained from the pre-treatment of bagasse from sugarcane by means of steam explosion followed by enzymatic hydrolysis of using the enzyme Cellic CTec3 (Novozymes).

The hydrolysate was characterized by Bazoti et al. (2017): 45.71 g.L^−1^ glucose, 28.5 g.L^−1^ of xylose, 6.32 g.L^−1^ of cellobiose, 1.05 g.L^−1^ of arabinose and fermentation inhibitors: 9.18 g.L^−1^ of acetic acid, 0.36 g.L^−1^ of furfural and 0.17 g.L^−1^ of hydroxymethylfurfural (HMF), in addition to an acid pH of 4.88. To assess the influence of detoxification (removal of acetic acid) in the production of second generation ethanol, the detoxify hydrolysate was through the process of stripping as experimental setup described in section “stripping” and then used as substrate in alcoholic fermentation. As control, non-detoxified (without removal of the acetic acid) hydrolysate was also employed as substrate.

#### Microorganism

The yeast with usual name of UFFS C.E.3.1.2 used in fermentation was recently isolated from the Brazilian ecosystem and described by Bazoti et al. (2017). The strain represents a new species of *Wickerhamomyces* (GenBank access number MF538579 and MF538580).

#### Alcoholic fermentation procedure

The yeast was maintained in yeast extract peptone dextrose (YPD medium - 1% yeast extract, 2% peptone, 2% glucose and 2% of agar). The growth of yeast occurred at 30°C in solid medium, after 72 h, it was transferred to the liquid medium, where it remained for over 24 h. The inoculum was then poured in sterile hydrolysate.

The detoxified hydrolysate sugarcane bagasse was diluted 1:3 (v/v), dilution determined after the completion of preliminary experiments (data not shown) and 90 mL of it were added in 250 mL erlenmeyer flask and sterilized in autoclave at 120°C for 15 min (Bazoti et al., 2017).

The fermentation was carried out in an orbital shaker at 30°C and 50 rpm in micro-aerophilic condition. The samples were taken every 24 h and the concentrations of glucose, xylose, cellobiose, arabinose, furfural, acetic acid, hydroxymethylfurfural (HMF) and ethanol has been measured.

These compounds were quantified by HPLC (Shimadzu chromatograph) equipped with a refractive index detector RID 10-A and an AMINEX® BIORAD HPX87H column. Samples of 20 μL were chromatographed at 45°C, with 5 mM H_2_SO_4_ as mobile phase, and flow rate of 0.6 mL/min. Furfural and HMF compounds (samples of 20 μL) were determined using a PDA 10-A detector operated with a C18 column, eluted with 1:8 acetonitrile/water and 1% acetic acid, at 30°C, and a flow rate of 0.8 mL/min. Before HPLC analyses, the samples were pre-filtered and diluted appropriately. The compounds concentration was determined by using calibration curves for each compound as analytical methodology proposed by Bazoti et al. (2017).

## Results and discussion

### Adsorption kinetics

Several mechanisms can control the adsorption process of the adsorbate on the adsorbent, they are: mass transfer, diffusion control, chemical reactions and particle diffusion (Mohan and Gandhimathi, 2009). For kinetic study, methods like Pseudo first-order, Pseudo second-order, and Intraparticle diffusion were employed in order to gauge the data obtained. Equation 1 and 2 describe the models for Pseudo first-order and Pseudo second-order according to Al-Othman et al. (2012), where: q_e_ is the concentration at equilibrium (mg.g^−1^); *t* is the adsorption time; q_t_ (mg.g^−1^) is the adsorption capacity at *t* (min); *k*_1_ (min^−1^) and *k*_2_ (g.mg^−1^.min^−1^) are the adsorption constants for Pseudo first and second-order, respectively. Table 1 presents the equilibrium concentration attained at the end of each kinetic in the solid phase, the coefficient of determination (*R*^2^) and the constants *k*_1_ and *k*_2_ for each method applied.

**Table 1:** Pseudo-first and Pseudo-second order adjusting data.

**Acetic Acid Concentration(%)**	**q_e_ (mg/g)**	**Pseudo first-order**		**Pseudo second-order**	
		**R^2^**	**k_1_ (min^−1^)**	**R^2^**	**k_2_ (g.mg^−1^.min^−1^)**
5	159.67	0.89	0.098	0.93	0.003
10	211.85	0.97	0.183	0.98	0.004
20	224.82	0.71	0.052	0.98	0.003

**Figure 2:**
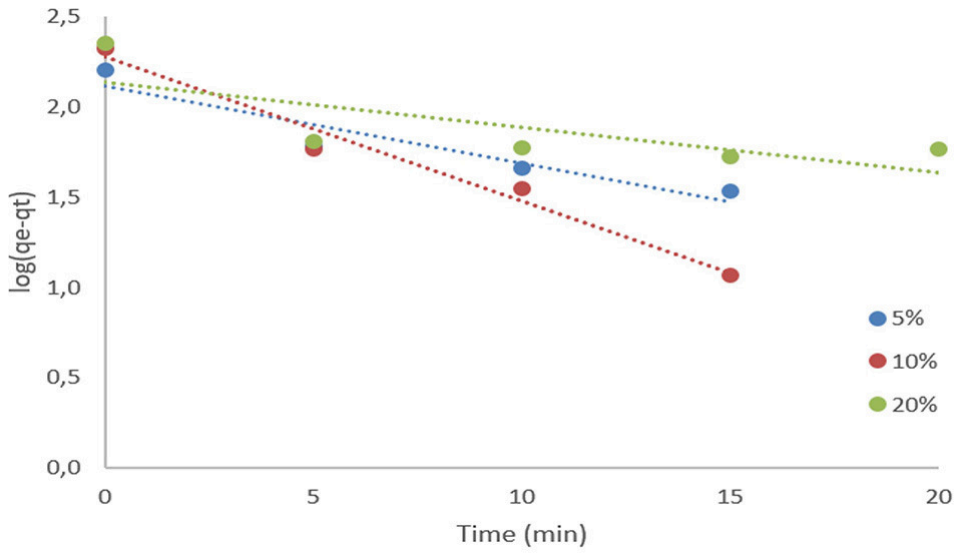
Pseudo-first order kinetic results.

**Figure 3:**
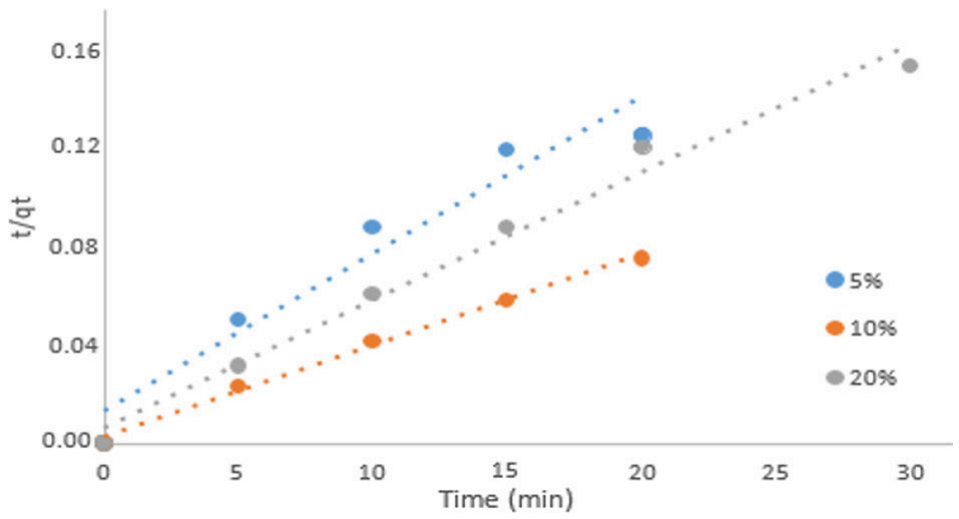
Pseudo-second order kinetic results.

Due to higher *R*^2^ outcomes, Pseudo second-order model showed a better fit to the adsorption process in the system, so it was assumed to be the second-order reaction the relevant for the process. Results suggest that a chemical interaction occurred between the surface of activated carbon and the component acetic acid, in other words, a chemical adsorption took place (Xu et al., 2013). Figures 2, 3 present the plot of the first-order and second-order models for adsorption of acetic acid at each concentration in analysis.

(1) $$\begin{array}{rcl} log\;\left(q_{e}-\;q_{\text{t}}\right) & = & logq_{e}-\;\frac{1}{2.303}\;k_{1}\text{t} \end{array}$$

(2) $$\begin{array}{rcl} \frac{t}{q_{t}} & = & \;\frac{1}{k_{2{q_{e}}^{2}}}+\;\frac{1}{q_{e}}\;t \end{array}$$

The limiting step of an adsorption process can be related to a mechanism of slow intraparticle diffusion, even considering an instantaneity sorption on the outer surface (Ho and McKay, 1998). This process can be dictated by two or more limiting phases: the first one is fast and occur on the adsorbent surface; the second one is a gradually adsorption, where the diffusion within the particle regulates the process; and the third one is at final step, where the intraparticle diffusion decreases and reaches to equilibrium (Chen et al., 2003). Equation (3) gives the behavior of the adsorption.

(3) $$\begin{array}{r} q_{t}=\;k_{in}\;t^{\frac{1}{2}} \end{array}$$

Where *k*_*in*_ (mg.g^−1^.h^−1/2^) is the constant for the intraparticle diffusion velocity. Table 2 presents data after employing Equation (3). It was possible to define the first as the regulating phase due to its higher *R*^2^ values. Table 2 also shows the constant for the intraparticle diffusion velocity for each experimental condition.

**Table 2:** Intraparticle diffusion adjusting data.

**Acetic acid Concentration(%)**	**First phase**		**Second phase**	
	***k_*in*_* (mg.g^−1^.*h*^−1/2^)**	***R*^2^**	***k_*in*_* (mg.g^−1^.*h*^−1/2^)**	***R*^2^**
5	37.54	0.97	34.46	0.90
10	80.53	0.96	18.23	0.94
20	55.92	0.93	16.36	0.72

### Adsorption isotherms

Considering an adsorption process, the isotherms are used to set thermodynamic characteristics between liquid and solid phases at an equilibrium condition (Maneerung et al., 2016). The Langmuir adsorption isotherm is based on the assumption that the adsorbate sorption takes place on the monolayer adsorption, and the process occur in a limited number of available sites. So, the equilibrium point is attained when no more sorption can occur (Allen et al., 1988). Hence the model can be represented by Equation (4), where *qmax* (mg.g^−1^) represents the maximum capacity in the monolayer, *bL* (L.g^−1^) represents the affinity between adsorbate and adsorbent, *qe* (mg.g^−1^) and *Ce* (g.L^−1^) are the concentration of solute in the solid and in the liquid phase at equilibrium, respectively.

(4) $$\begin{array}{r} q_{e}=\;\frac{q_{\max}\;b_{L\;}C_{e}}{1+\;b_{L\;}C_{e}} \end{array}$$

Furthermore, adsorption process may also be characterized by a dimensionless parameter named separation factor (R_L_) expressed by Equation (5), where C_0_ is the initial concentration of the adsorbate. This parameter infer if the adsorption is unfavorable (R_L_ > 1), linear (R_L_ = 1), favorable (0 < R_L_ < 1) or irreversible (R_L_ = 0) (Webber and Chakkravorti, 1974).

(5) $$\begin{array}{r} R_{L}=\;\frac{1}{1+\;b_{L\;}C_{0}} \end{array}$$

Supposing that the relation between the quantity of adsorbate retained by the adsorbent and the concentration of adsorbate in the solution is not a constant for different solution concentrations, the Freundlich adsorption model suggests that if the concentration of adsorbate in the medium at equilibrium *Ce* was augmented to the power of *n*, the quantity of material adsorbed would be *qe*, and the relation between then would be a constant value at a certain temperature (Allen et al., 1988). Thus, the empirical Equation (6) is given, where *k*_*F*_ and *n* are the Freundlich constant and exponent, respectively.

(6) $$\begin{array}{r} q_{e}=\;k_{F}\;{C_{e}}^{\frac{1}{n}} \end{array}$$

The Freundlich model considers that adsorption occur onto multilayers and describes better adsorption on heterogeneous surfaces (Fritz et al., 1981). The reaction is dictated as favorable if the exponent *n* is situated between the range 1 and 10.

**Figure 4:**
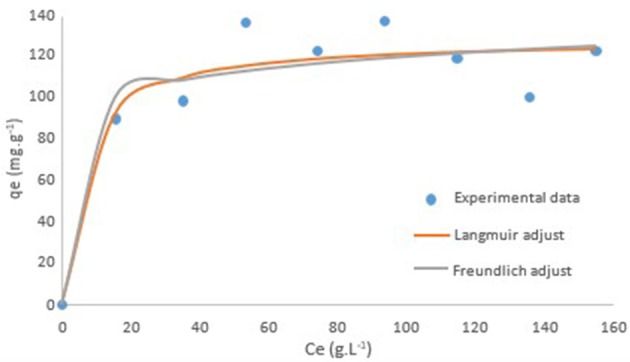
Langmuir and Freundlich adjusts over experimental data.

Outcomes from isotherm experiments carried out at 20, 40, 60, and 70°C presented an unclear distinction among the temperatures in study. A statistical analysis was conducted and no significant difference was found (data not shown), leading to the idea that temperature is not a variable for the adsorption of acetic acid by activated carbon. Thus, once changes in temperature were evaluated as not significant for the process, an average was taken from all runs at each concentration. Modeling procedure according to Langmuir and Freundlich methods was employed in order to adjust the data from the isotherm composed. Two models, one for each method, were built and are presented in Figure 4.

Parameters defined by Software STATISTICA 8.0 for both adjusts, Langmuir and Freundlich, are shown in Table 3. Similar values for *R*^2^ was found, but a higher value suggests that Langmuir model fits experimental data better than Freundlich model. The value of *qmax* of 128.66 mg.g^−1^ dictate the maximum capacity of the activated carbon on retaining acetic acid, and the result for calculated with the lower used in the isotherms was 0.30 indicating a favorable adsorption.

**Table 3:** Parameters for Langmuir and Freundlich isotherms adjusting.

	**Parameters**	**Estimate**	**Standard error**	***p*-level**	***R*^2^**
Langmuir	*Qmax*	128.66 mg.g^−1^	9.09	0.000002	0.90
	b_L_	0.16 L.g^−1^	0.10	0.165699	
Freundlich	K	76.94	23.12	0.012649	0.88
	N	10.39	7.41	0.203635	

Singh and Kumar (2016) employed the Langmuir isotherm model to evaluate adsorption of CO_2_ onto activated carbon in granular form, the maximum adsorption range was found as 483.55 and 364.22 mg.g^−1^ at 25° and 65°C as temperature with *R*2 varying between 0.94 and 0.91. Maneerung et al. (2016) found a *qmax* of 189.8 mg.g^−1^ applying the same modeling method for the adsorption pair char/rhodamine B. Li et al. (2016) used H_3_PO_4_ as activated agent on carbon engendered from *Eupatorium adenophorum*, the Langmuir isotherm model gave a maximum adsorption of 351.0 mg.g^−1^ of congo red onto this adsorbent.

### Stripping

**Figure 5:**
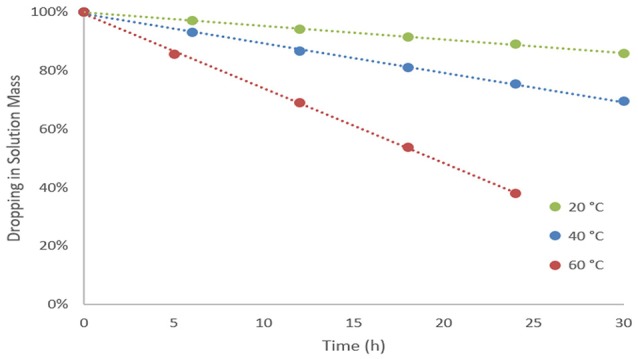
Dropping in solution mass for each kinetic.

Figure 5 presents results for dropping in mass during stripping process and the influence when temperature variation takes place. After 30 h, mass dropping in the solution was quantified as 86 and 69% for running at 20° and 40°C, respectively. For temperature 60°C, it showed to be impracticable to continue experiments after 24 h due to volume losing, at that time solution mass was 38% from the beginning. Linear equations with *R*^2^ values higher than 0.99 were taken from data and coefficients for kinetics at 20, 40, and 60°C presented to be 0.46, 1.01, and 2.55 (%.h^−1^), respectively.

Concentration values on liquid presented no significant variation during the kinetic, indicating air is not efficient on promoting an increment on removal of the volatile component and concentrating water in the medium. Acetic acid removal from the solution was quantified. Results given in milligrams of acetic acid removed per liter of air employed are 1.98, 4.24, and 12.32 for the kinetics conducted at 20, 40, and 60°C, respectively. This results shows the influence of temperature on the process, augmenting outcomes considerably.

### Ethanol production

After the fermentation using detoxified hydrolysate, from the process of stripping, an ethanol concentration of 8.3 g.L^−1^ was obtained, after 72 h of fermentation. The results of the fermentation showed that after 48 h of fermentation with the detoxified hydrolysatedetoxified hydrolysate, the ethanol concentration reached 7.4 g.L^−1^, while the fermentation performed with the non-detoxified hydrolysate reached only 1.1 g.L^−1^ at the same time. This indicates that with the removal of inhibitory components the yeast adapt more easily to the middle and consequently managed to convert more quickly those carbohydrates into ethanol.

The concentration of ethanol obtained was significantly greater than values obtained in previous studies. Ferreira et al. (2011) obtained, from detoxified hydrolysate, a concentration of 2.7 g.L^−1^ after 48 h of fermentation with *Scheffersomyces stipitis* UFMG-IMH 43.2. Gutiérrez-Rivera et al. (2015) reported the production of 2.26 g/L of ethanol from sugarcane bagasse hydrolysate of raw sugar, in the presence of 4.5 g.L^−1^ of acetic acid fermentation by *S. stipitis* NRRL Y-7124.

Still, Gutiérrez-Rivera et al. (2015) affirm that the increase in the percentage of the hydrolysate causes a decrease in the production of ethanol. This occurs due to the increase in the concentration of inhibitory components in the medium, such as acetic acid, HMF and furfural, suggesting a negative impact on the activity of fermentation in the presence of inhibitory components. This impact was also observed in our study, emphasizing the importance and the potential for application of the process of stripping for removal of inhibitory components of the fermentation medium.

## Conclusion

Determining adsorption kinetics and adsorption isotherms is essential for evaluating physical and thermodynamic properties between pairs of adsorbate-adsorbent. Pseudo-second order model showed to greatly fit to the data and define the adsorption reaction as a second order one, indicating that a chemical interaction took place during adsorption process. The Langmuir isotherm presented to better describe experimental data when compared to Freundlich model, demonstrating a homogeneous adsorption onto the activated carbon in test. Parameters from the Langmuir model infers that the adsorption is favorable and indicates the value of 128.66 mg.g^−1^ as the maximum adsorption capacity between the pair in study. For stripping process, increases in temperature improves considerably acetic acid volatilization, but it was not effective on concentrating water in the medium. However, the stripping process proved to be efficient for the removal of inhibitory components of the sugarcane bagasse hydrolysate, increasing ethanol production from 1.1 g.L^−1^ to 7.4 g.L^−1^ in the alcoholic fermentation process.

## Author contributions

WA and EB: experimental procedures, CB: alcoholic fermentation, SB: analytical quantification, AD: adsorption modeling, SA: yeast maintenance, HT: research coordinator.

### Conflict of interest statement

The authors declare that the research was conducted in the absence of any commercial or financial relationships that could be construed as a potential conflict of interest.
